# Biomarkers Predict Relapse in Granulomatosis with Polyangiitis

**DOI:** 10.1155/2014/596503

**Published:** 2014-04-30

**Authors:** Patrick C. P. Hogan, Robert M. O'Connell, Simone Scollard, Emmett Browne, Emer E. Hackett, Conleth Feighery

**Affiliations:** ^1^Department of Immunology, Trinity College Dublin, St. James's Hospital, Dublin 8, Ireland; ^2^Institute of Molecular Medicine, Trinity College Dublin, St. James's Hospital, Dublin 8, Ireland

## Abstract

Granulomatosis with polyangiitis (GPA) is a small blood vessel vasculitic disorder with a high mortality rate if undiagnosed or treated inadequately. Disease relapse is a key feature of this disease and early identification of relapse episodes is very important in limiting end-organ damage. The advent of indirect immunofluorescence to detect antineutrophil cytoplasmic antibody (ANCA) with specific reactivity against the enzyme proteinase-3 (PR3) has been very useful in the diagnosis of GPA but is less helpful in predicting relapse. Indeed, up to date no satisfactory biomarker has been identified that can reliably predict relapse. This study assessed the probability of the occurrence of a relapse when a change was noted in a range of commonly used laboratory tests. These tests included levels of C-reactive protein (CRP), anti-PR3 antibodies, ANCA titre, and the neutrophil count. A group of 30 GPA patients with a total of 66 relapse episodes was investigated and a novel clinical yield score was devised. When a combined rise in CRP, anti-PR3 antibodies, and neutrophil count was observed in the 6-month period before a relapse event, 59% of patient relapses could be predicted. Monitoring changes in this set of parameters helps identify disease relapse.

## 1. Introduction

Granulomatosis with polyangiitis (GPA) is an autoimmune vasculitis primarily affecting the respiratory tract and kidneys and is characterised by necrosis and granulomatous lesions involving small to medium sized blood vessels. The principal autoantibody associated with the disease is the antineutrophil cytoplasmic antibody (ANCA), usually directed against the enzyme proteinase-3 (PR3) [1, 2].

Long term survival in GPA has greatly improved since the introduction of disease modifying agents such as cyclophosphamide, with a ten-year survival of approximately 75% [3]. Disease relapse is a major cause of morbidity and mortality in patients with GPA, with some 50% of patients experiencing relapse at five-year followup [4]. Prediction of disease relapse is therefore an important goal of clinical management. Certain patient and disease factors have been identified as increasing the risk of relapse. For example, it has been shown that lung involvement, infection with* staphylococcus aureus*, and cardiac or renal involvement increase the chance of relapse [5–7].

The identification of biomarkers that predict relapse in GPA has proven elusive. While a change in ANCA titre may be associated with disease activity, it is not sufficiently specific nor sensitive to be an effective predictor of relapse [8] and hence is considered to be no more than moderately useful [9, 10]. For example, if a rise in ANCA titre alone was used as an indicator of disease relapse, this could expose patients to unnecessary and potentially toxic effects of immunosuppression [11]. No role in the prediction of relapse has been found for conventionally measured inflammatory markers such as C-reactive protein (CRP) or the erythrocyte sedimentation rate [12].

The purpose of this study was to examine whether a combination of commonly measured laboratory parameters might help predict disease relapse in patients with GPA. A novel clinical yield formula was devised to examine changes in the identified biomarkers in the six-month period before the relapse event.

## 2. Methods

Patients attending St. James's Hospital who had been diagnosed with GPA between 1984 and 2012 were included. The study was conducted using patients' medical charts and the hospital investigation database. Patients were required to have had evidence of one or more major relapse events to be eligible for inclusion. A major relapse was defined as the commencement of potent immunosuppressive therapy, such as cyclophosphamide or rituximab, together with high dose prednisolone. Disease relapse was distinguished from an intercurrent infection based on the clinical judgment of a consultant clinical immunologist. Patients were also required to have a granular, cytoplasmic staining pattern on ANCA indirect immunofluorescence (IIF) and to be anti-PR3 antibody positive on enzyme-linked immunosorbent assay (ELISA). Test results for eight biomarkers were collected for each patient throughout followup: ANCA titre, anti-PR3 antibody level, CRP and creatinine levels, white cell count, neutrophil count, lymphocyte count, and monocyte count.

For ANCA IIF, serum samples were diluted 1/20 and examined using INOVA neutrophil coated slides. Anti-PR3 antibody levels were measured using the Biodiagnostics ELISA system. Full blood and differential white cell counts were performed on automated equipment (Sysmex XE-2000) and creatinine levels assayed using the Hitachi-747 analyser. CRP values were measured using the Behring BN2 nephelometer.

For each biomarker, a mean remission baseline value was calculated in order to exclude results which coincided with periods of disease activity. Hence, the following test results were excluded in calculating this remission baseline: all results from the twelve-month period after diagnosis and results from the six-month period before and the six-month period after the occurrence of a relapse. Having excluded these results, the remaining test results were termed as the remission baseline value. The baseline values were then used to calculate a “clinical yield” according to the following formula, in which the “prerelapse period” is defined as the six-month period prior to relapse:

 Clinical Yield = ((Number of Results (elevated or decreased) in pre-relapse period)/(Number of Results (elevated or decreased) in remission and pre-relapse period)) × 100.

The clinical yield formula has been previously used to evaluate the usefulness of other diagnostic techniques [13]. The value of this method of analysis is that it gives a true representation of the usefulness of tests, since it includes all the patients' test results as they were carried out in the clinical setting. Statistical significance was calculated using the Student's* t*-test.

Statistically significant results were combined to produce a composite clinical yield value for concurrent elevations in these biomarkers. White cell count was omitted from this calculation to prevent duplication of results of the neutrophil count.

## 3. Results and Discussion

In total 52 patients were identified for participation in this study. Of these, 22 patients were excluded from the study; 12 had never had a relapse; and a further 10 had inadequate test results. The characteristics of the remaining cohort of 30 patients are shown in Table 1. These 30 patients experienced a total of 66 relapses events. The total number of test results from these patients was 8,376. This was composed of 716 ANCA tests; 494 anti-PR3 antibody tests; 1,212 CRP measurements; and 1,198 white cell counts with a similar number of neutrophil, monocyte, and lymphocyte counts. 1,176 creatinine tests were also evaluated. The clinical yields of these biomarkers are shown in Table 2.

The clinical yield for all the biomarkers was greater for elevated results in comparison to decreased levels. For example, the results show a clinical yield of 32.31% for an elevation in CRP when compared to baseline values and 15.67% for a decrease in CRP. This means that the finding of an elevation in the CRP level was followed by a relapse within 6 months in 32.31% of cases but not in the remaining 67.69% of such cases. Likewise, when CRP values were lower than baseline values, this decrease preceded a relapse in 15.67% of cases.

The clinical yield for changes in white cell count, neutrophil count, CRP, and anti-PR3 antibody values was each statistically significant (Table 2). When the results for the neutrophil count, CRP, and anti-PR3 antibodies were examined as a group, the clinical yield was found to be 58.89% (*P* = 0.02). Thus, from a clinical perspective, a relapse could be predicted to develop within 6 months in some 59% of such cases. This finding matches clinical experience; for example, when a patient demonstrates an evolving rise in CRP, anti-PR3 antibodies, and neutrophil count, this is frequently accompanied by clinical deterioration and eventual relapse. Nonetheless, reliance on these three parameters would fail to indicate the development of relapse in 41% of cases. Further information can be gleaned from the situation where there is a fall in the levels of these parameters, when relapse is very unlikely and in 85% of such cases, patients are predicted to remain relapse-free. Finally, the results showed no significant predictive value for ANCA titres, serum creatinine levels, monocyte counts, or lymphocyte counts, when examined alone (Table 2).

## 4. Conclusions

After clinical remission has been induced in a patient, the common practice is to reduce and if possible to stop immunosuppressive treatment entirely. However, because disease relapse is so common, developing in 50% of patients [4], it is important to identify relapse early, so further treatment with immunosuppression can be introduced [14, 15]. However, clinicians are very mindful of the potential toxic side effects of this therapy [16]. On occasions, secure identification of a relapse is difficult. Hence, clinical details and laboratory results are carefully evaluated to determine if a relapse is taking place. Numerous studies have assessed the usefulness of ANCA in relapse prediction in GPA. A recent meta-analysis of such trials showed a link between a rise or persistent elevation in ANCA and relapse. However, the authors of this meta-analysis acknowledge that their results show only a modest correlation [9]. The findings of the present study show that when a rise is observed in the combination of the neutrophil count, CRP levels, and anti-PR3 antibodies, this is useful adjunctive evidence of relapse. This combination was found to be more strongly associated with relapse than any biomarker alone. However, there is still a need to develop improved biomarkers of disease activity in this life-threatening inflammatory disorder.

## Figures and Tables

**Table 1:** Clinical details of 30 patients with GPA with major disease relapse events.

Characteristic	Cohort
Total number of patients	30
Male	15 (50%)
Female	15 (50%)
Mean age at diagnosis (years)	47.57 (±16 years)
Mean length of followup (years)	11.73 (±5 years)
Mean age at first relapse (years)	52.1 (±16 years)
Mean time from diagnosis to first relapse (years)	4.53 (±5 years)
Total number of relapse events	66
Mean number of relapses per patient	2.2

**Table 2:** Clinical yield of a series of relapse biomarkers in 30 patients with GPA.

Biomarker	Clinical yield if <baseline	Clinical yield if >baseline	*P* value
White cell count	11.22%	30.21%	0.01
Neutrophil count	11.98%	31.43%	0.01
Lymphocyte count	18.13%	23.53%	0.92
Monocyte count	20.23%	21.40%	0.63
ANCA titre	18.24%	22.79%	0.7
Anti-PR3 antibodies	7.86%	24.81%	0.0003
CRP	15.67%	32.31%	0.002
Creatinine	17.33%	23.40%	0.74
Neutrophil count, CRP, and anti-PR3	14.81%	58.89%	0.02
